# Analgesic Neural Circuits Are Activated by Electroacupuncture at Two Sets of Acupoints

**DOI:** 10.1155/2016/3840202

**Published:** 2016-06-27

**Authors:** Man-Li Hu, Zheng-Ying Qiu, Kuang Hu, Ming-Xing Ding

**Affiliations:** ^1^College of Veterinary Medicine, Huazhong Agricultural University, Wuhan 430070, China; ^2^College of Life Science and Technology, Huazhong Agricultural University, Wuhan 430070, China

## Abstract

To investigate analgesic neural circuits activated by electroacupuncture (EA) at different sets of acupoints in the brain, goats were stimulated by EA at set of Baihui-Santai acupoints or set of Housanli acupoints for 30 min. The pain threshold was measured using the potassium iontophoresis method. The levels of c-Fos were determined with Streptavidin-Biotin Complex immunohistochemistry. The results showed pain threshold induced by EA at set of Baihui-Santai acupoints was 44.74% ± 4.56% higher than that by EA at set of Housanli acupoints (32.64% ± 5.04%). Compared with blank control, EA at two sets of acupoints increased c-Fos expression in the medial septal nucleus (MSN), the arcuate nucleus (ARC), the nucleus amygdala basalis (AB), the lateral habenula nucleus (HL), the ventrolateral periaqueductal grey (vlPAG), the locus coeruleus (LC), the nucleus raphe magnus (NRM), the pituitary gland, and spinal cord dorsal horn (SDH). Compared with EA at set of Housanli points, EA at set of Baihui-Santai points induced increased c-Fos expression in AB but decrease in MSN, the paraventricular nucleus of the hypothalamus, HL, and SDH. It suggests that ARC-PAG-NRM/LC-SDH and the hypothalamus-pituitary may be the common activated neural pathways taking part in EA-induced analgesia at the two sets of acupoints.

## 1. Introduction

Electroacupuncture (EA), a widely used version of acupuncture which provides a stimulating current to acupoints through acupuncture needles, is effective and quantifiable. Electroacupuncture-induced analgesia (EAA) has been applied to ameliorate pain not only in varieties of painful diseases [1–4], but also in various operations, such as cesarean section, gastrectomy, enterectomy, and castration in humans or animals during the 1970s [5–7]. Some researchers used classical physical approaches (the stimulation and abolition of certain nerve fibres or nuclei) as well as pharmacological approaches (assessing antagonists and antibodies) to identify that EAA is involved in some cerebral nuclei or areas such as the nucleus amygdala (AMY), the periaqueductal grey (PAG), and the locus coeruleus (LC) [8–12]. Because nuclei are linked to each other with their fibres and constitute a complex network, the function of a single nucleus or area cannot elucidate the neural mechanism underlying EAA. Functional magnetic resonance imaging (fMRI) has been used to explore the cerebral regions activated by EA. Chiu et al. [13] found a tendency for the activation of pain-modulation areas induced by EA at bilateral Hegu or Zusanli acupoints was prominent as compared with EA stimulation at bilateral Neiguan acupoints in rats through fMRI. Similarly, it has been demonstrated that EA at different acupoints can elicit different fMRI-activated patterns in the human brain [14, 15]. These studies indicate that the central activated regions are acupoint-dependent. However, EAA-related neural circuitries activated by EA at different sets of acupoints are not clear yet.

C-fos, an immediate early gene, is expressed at low levels in the intact brain under basal conditions and responses rapidly and transiently after various stimulations, including restraint [16, 17], noise [17, 18], and pain [19]. Its protein product, Fos, as a marker, has been used to reveal functional heterogeneity among neuronal subpopulations [20] and map neuronal pathways in the central nervous system (CNS). In addition, c-Fos protein is a part of transcription factor AP-1 involved in the regulation of target genes when cells become activated. The verified target genes of c-Fos include active substances participating in EAA in the CNS, such as preproenkephalin [21, 22], proopiomelanocortin [23, 24], preprodynorphin [25], 5-TH receptors [26], and cholecystokinin [27].

It has been demonstrated that EAA varies in species [28–30]. These studies have shown that EA in combination with analgesics reduces the required dose of analgesics in humans [30], rats [29], and goats [28] by 48%, 50%, and over 75%, respectively. It is clear that the analgesic effect induced by EA in goats (ruminants) is superior to that in humans or rats if the species or the analgesic properties are not considered. Ruminants can be optimal model animals for investigating the mechanisms underlying EAA. In the present study, to explore central neural mechanism by which EA induces analgesia, goats were stimulated by EA at two sets of acupoints, and the expression levels of c-Fos were measured in analgesia-related nuclei or areas, including the caudate nucleus (CAU), the nucleus accumbens (ACB), the lateral septal nucleus (LSN), the medial septal nucleus (MSN), the paraventricular nucleus of the hypothalamus (PVH), the ventromedial nucleus of the hypothalamus (VMH), the arcuate nucleus (ARC), the nucleus amygdala basalis (AB), the lateral habenula nucleus (HL), the caudal ventrolateral periaqueductal grey (vlPAG), the parabrachial nucleus (PBN), LC, the nucleus raphe magnus (NRM), the gigantocellular reticular nucleus (GI), the nucleus tractus solitarius (NTS), the anterior lobe of the pituitary gland (PG), and spinal cord dorsal horn (SDH). It is hypothesized that there exist common neural circuits among the analgesia-related nuclei or areas activated by sets of acupoints.

## 2. Materials and Methods

### 2.1. Animals

One-year-old healthy crossbred male goats (30 ± 2 kg body wt, *n* = 28) were purchased from Hubei Agricultural Academy of Science. The goats were randomly assigned for 4 treatments: the blank control, the sham control, EA at Baihui and Santai acupoints, and EA at bilateral Housanli acupoints, 7 goats per group. The goats were housed in the same management condition with food and water ad libitum. A quiet environment was provided, and the room temperature was maintained 22 ± 2°C during experiment. Experimental animals were accustomed to being approached and restrained (1 h/day) for two weeks before the start of the experiment. The experimental protocol (HZAUGO-2015-005) was approved by the Animal Care Center, College of Veterinary Medicine, Huazhong Agricultural University (Wuhan, China).

### 2.2. Electroacupuncture Procedures

For EA stimulation, the set of Baihui and Santai acupoints and the set of bilateral Housanli acupoints (equal to Zusanli in humans) were selected for two EA groups. Here the acupoints are nominated with Pinyin Naming System instead of the Meridian Numbering System because animal's meridians are not completely recorded. The anatomical locations of these acupoints have been described in detail for use in veterinary medicine [28, 31–33]. The Baihui acupoint was identified on the dorsal midline between the spinous processes of the last lumbar and the first sacral vertebrae. The Santai acupoint was identified on the dorsal midline between the spinous processes of the fourth and fifth thoracic vertebrae. The Housanli acupoint was identified in the muscular groove between the long digital extensor and the lateral digital extensor muscles below the head of the fibula on the bilateral lateral surface of the bilateral legs. The needles were disinfected and inserted into the acupoints by a skilled acupuncturist after acupoint sites were shaved and disinfected with 75% ethanol. A stainless steel acupuncture needle (0.40 mm in diameter and 50 mm in length) was perpendicularly inserted into the Baihui acupoint to a depth of approximately 30 mm. For the Santai acupoint, a needle (0.45 mm in diameter and 75 mm in length) was inserted into the acupoint to a depth of 40 to 50 mm at a 45° angle. For bilateral Housanli acupoints, two needles (0.40 mm in diameter and 50 mm in length) were perpendicularly inserted into each point to a depth of 20 to 30 mm. The needles in each set of EA group were connected by a pair of wires to one output of WQ-6F Electronic Acupunctoscope (Beijing Xindonghua Electronic Instrument Co., Ltd., Beijing, China). According to Cheng et al. [33], the goats were treated with EA of 60 Hz and 3.2 V for 30 min. Needles were inserted into Baihui and Santai acupoints of the goats without electricity as the sham control. The goats were restrained in the same manner as EA-stimulated goats without needling and EA as the blank control.

### 2.3. Determination of Pain Threshold

Pain threshold was assessed immediately before and after EA administration by one skilled person who was blinded to the goat assignments. The pain was induced by potassium iontophoresis [28, 34, 35] passing through the skin by a direct current induction therapy apparatus (Shantou Medical Equipment Factory Co., Ltd., Shantou, China). The site was shaved and cleaned with soap and water and sterilized with 75% ethanol. Two electrodes were soaked with saturated potassium chloride and placed 1-2 cm apart on the center of the left flank skin. The pulse direct current delivered potassium ions into the subcutaneous tissues while voltage was continuously increased. When obvious contraction of the local skin and muscle along with head turning toward the abdomen, back hunching, and body eluding movement were observed, the voltage level was recorded. The procedure was repeated three times with 5 min interval. Mean voltages before and after EA were expressed as *V*_0_ and *V*_*n*_, respectively. The change of percentage in pain threshold was calculated by the following formula: Δ% = (*V*_*n*_ − *V*_0_)/*V*_0_ × 100%.

### 2.4. Immunohistochemistry

**Figure 1:**
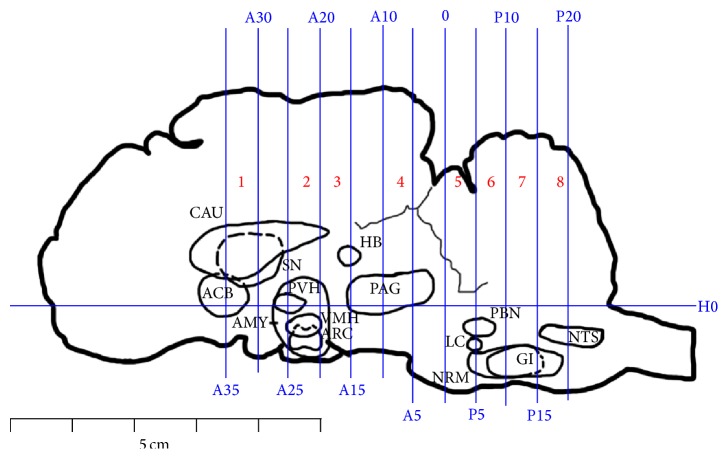
Brain sectioning. H0: the horizontal zero plane. A35, A30, A25, A20, A15, A10, A5, and 0 show transverse planes at 35, 30, 25, 20, 15, 10, 5, and 0 mm rostral to the interaural line, and P5, P10, P15, and P20 show transverse planes at 5, 10, 15, and 20 mm caudal to the interaural line, respectively. The nuclei and areas identified include the nuclei or areas to be observed were the caudate nucleus (CAU), the nucleus accumbens (ACB), the septal nucleus (SN), the paraventricular nucleus of the hypothalamus (PVH), the ventromedial nucleus of the hypothalamus (VMH), the arcuate nucleus (ARC), and the amygdala (AMY), the nucleus habenula (HB), the periaqueductal grey (PAG), the parabrachial nucleus (PBN), the locus coeruleus (LC), the nucleus raphe magnus (NRM), the gigantocellular reticular nucleus (GI), and the nucleus tractus solitarius (NTS).

The levels of c-Fos protein were measured through the method of SABC immunohistochemistry. Once the pain threshold was measured after EA, experimental goats were deeply anesthetized with intravenous administration of xylidinothiazoline at 3 mg/kg. Physiological saline was infused through bilateral carotid arteries while the blood flowed out from the jugular veins. When the liquid became transparent, 4% percent paraformaldehyde instead of the physiological saline was infused for 1 h. The pituitary gland, brain, and a part of the cervical spinal cord C1 were taken out of the skull and cervical vertebral canal. The brain was transected into 8 blocks (B 1–8) with the method described by Qiu et al. [31]. The vertical interaural plane represented the zero reference point for the anterior-posterior coordinates, and the horizontal zero plane (H0) intersected the interaural point and a point 25 mm above the lower margin of the orbit. Blocks B1–B8 are from anterior 35 mm (A35) to anterior 30 mm (A30), anterior 25 mm (A25) to anterior 20 mm (A20), anterior 20 mm (A20) to anterior 15 mm (A15), anterior 10 mm (A10) to anterior 5 mm (A5), 0 mm (0) to posterior 5 mm (P5), posterior 5 mm (P5) to posterior 10 mm (P10), posterior 10 mm (P10) to posterior 15 mm (P15), and posterior 15 mm (P15) to posterior 20 mm (P20), respectively (Figure 1).

**Figure 2:**
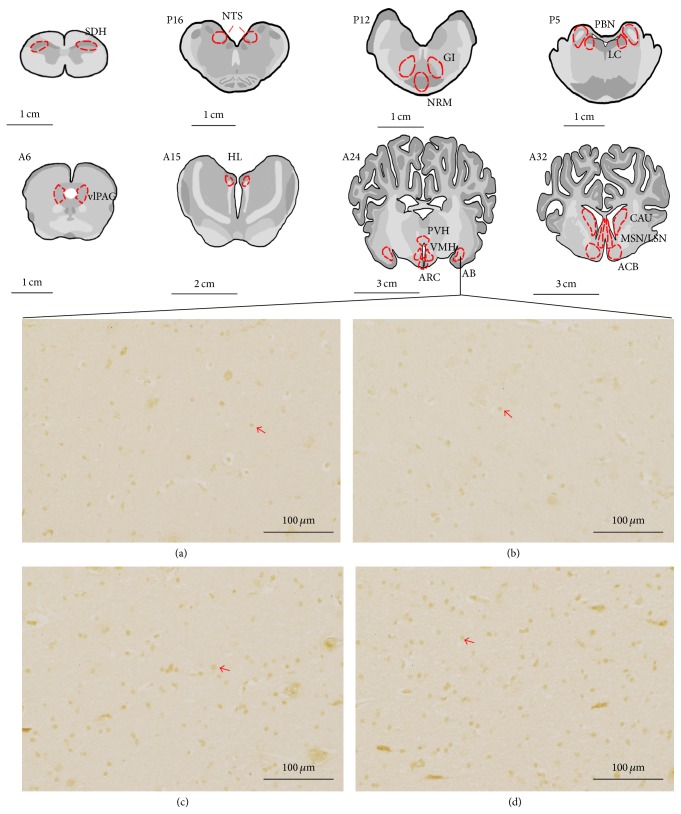
The nuclei (areas) locations used for the c-Fos-like immunoreactive (c-Fos-IR) neurons counts and the representative c-Fos-IR neurons in AB. C1: spinal cord dorsal horn (SDH). P16: NTS at interaural levels of −16 mm. P12: NRM and GI at interaural levels of −12 mm. P5: PBN and LC at interaural levels of −5 mm. A6: the ventrolateral periaqueductal grey (vlPAG) at interaural levels of 6 mm. A15: the lateral habenula nucleus (HL) at interaural levels of 16 mm. A24: PVH, VMH, ARC, and nucleus amygdala basalis (AB) at interaural levels of 24 mm. A32: ACB, CAU, the lateral septal nucleus (LSN), and the medial septal nucleus (MSN) at interaural levels of 32 mm. (a)–(d) show positive c-Fos-IR neurons in AB in the blank control, sham control, EA at the set of bilateral Housanli acupoints, and the set of Santai and Baihui acupoints, respectively. Arrows point to the positive neurons. The bars = 100 *μ*m.

The nuclei or areas were identified according to the brain atlas of goats and pigs and the morphological characteristics of the neurons [36–39]. The nuclei or areas to be observed were the CAU, ACB, LSN, and MSN in B1, the PVH, VMH, ARC, and AB in B2, the HL in the caudal part of B3, the vlPAG in B4, the PBN and the LC between the caudal part of B5 and the rostral part of B6, the NRM and GI in B7, and the NTS in B8 (Figure 1). The blocks were embedded in paraffin with their rostral surface facing up. Each of the blocks was consecutively sectioned with a thickness of 5 *μ*m. Four slides of each nucleus or brain area were mounted on polylysine coated slides, deparaffinized, and rehydrated sequentially. Among these four slides, three of them were incubated with rabbit-anti-c-Fos (Wuhan Boster Biological Technology Ltd., Wuhan, China; 1 : 50 diluted in PBS) while one was incubated with PBS instead of the antibody as the negative control. The remaining experimental procedures of SABC immunohistochemistry followed the instructions provided by the reagent company (Wuhan Boster Biological Technology Ltd., Wuhan, China). The nucleus of positive cells was stained as brown yellow. The locations of the observed nuclei (areas) with the representative stained cells are shown in Figure 2.

Optical images of the stained nuclei or area in the CNS were obtained under a light microscope (Nikon ECLIPSE 80I, Nikon Corporation, and Tokyo, Japan) connected to a video-based and computer-linked system (high-resolution pathological image analysis system 1000, Wuhan Qianping Ltd., Wuhan, China). Three slides of each nucleus or brain area were observed with 200x magnification on both sides. The number of c-Fos-like immunoreactive (c-Fos-IR) cells on each nucleus or brain area was counted by the Image-Pro plus 6.0 system (MediaCybernetics, Inc., Bethesda, MD, USA). The mean values calculated from each nucleus or brain area represented the c-Fos-IR cells per goat.

### 2.5. Statistical Analysis

All data were presented as the mean ± SD. Data analysis was performed with SPSS 18.0 software (SPSS Inc., Chicago, USA). The pain threshold and the number of c-Fos-IR cells were analyzed using one-way ANOVA, followed by Bonferroni's post hoc test. The value of *p* < 0.05 was considered to be significant.

## 3. Results

### 3.1. Analgesic Effects Induced by Electroacupuncture

**Figure 3:**
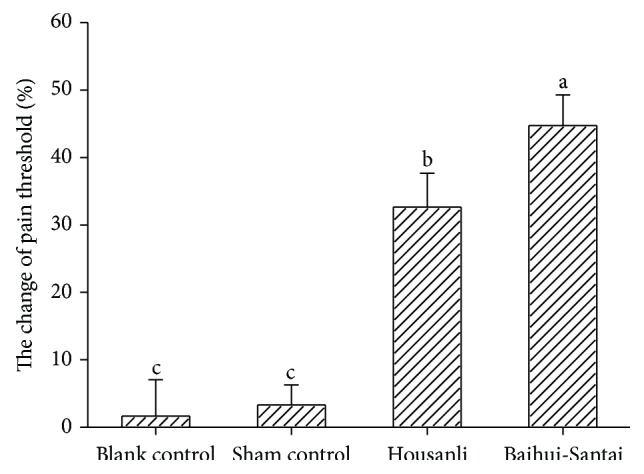
Analgesic effects induced by EA in goats (mean ± SD, %, *n* = 7). The pain thresholds were measured with potassium iontophoresis. EA significantly increased pain threshold. The values with different letters differ significantly (*p* < 0.05). The blank control refers to the group in which the goats were restrained as the EA-treated goats without needling and electric stimulation. The sham control refers to the group in which needles were inserted into Baihui and Santai acupoints of the goats without electric stimulation.

The EAA effects were represented by the changes in the pain threshold (Figure 3). No significant difference (*p* = 1.000) in the pain threshold was found between the blank control (1.64% ± 5.39%) and the sham control (3.29% ± 2.96%). Compared with the blank control, the pain threshold in the two EA groups increased (*p* < 0.05). The pain thresholds in goats treated with EA at the set of Baihui and Santai acupoints and at the set of bilateral Housanli acupoints increased by 44.74% ± 4.56% and 32.64% ± 5.04%, respectively, with the former significantly higher (*p* = 0.001) than the latter.

### 3.2. The Expression Levels of c-Fos Induced by EA

C-Fos-IR cells were observed in the analgesia-related nuclei and areas, which included CAU, ACB, LSN, MSN, PVH, VMH, ARC, AB, HL, vlPAG, PBN, LC, NRM, GI, NTS, PG, and SDH (Table 1). There was no difference (*p* > 0.05) in the number of c-Fos-IR cells between the blank control and the sham control. Compared with the blank control, c-Fos-IR cells in goats treated with EA at the set of bilateral Housanli acupoints increased (*p* < 0.05) in the LSN, MSN, PVH, VMH, ARC, AB, HL, vlPAG, PBN, LC, NRM, GI, PG, and SDH, while c-Fos-IR cells in goats receiving EA at the set of Baihui and Santai acupoints increased (*p* < 0.05) in the MSN, VMH, ARC, AB, HL, vlPAG, PBN, LC, NRM, GI, PG, and SDH. Compared with EA at the set of bilateral Housanli acupoints, EA at the set of Baihui and Santai acupoints induced c-Fos-IR cells to increase (*p* < 0.05) in the AB, but to decrease (*p* < 0.05) in the MSN, PVH, HL, and SDH.

## 4. Discussion

Numerous studies and clinical practices have shown that EA can induce potent analgesia. Wang et al. [40] used EA to stimulate bilateral Zusanli and Kunlun acupoints in rabbits and found that better analgesia was induced by 2 Hz, followed by 30 Hz > 60 Hz > 100 Hz. Wang et al. [41] reported that 100 Hz EA at bilateral Zusanli and Sanyinjiao acupoints induced more effective analgesia than 2 Hz in rats. Cao et al. [42] reported that 15 Hz EA at bilateral Yanglingquan acupoints induced more effective analgesia than 100 Hz EA in rats. In goats, Cheng et al. [33] compared analgesic effects induced by 0, 2, 40, 60, 80, and 100 Hz of EA for 30 min and found that 60 Hz produced more potent analgesia than any other frequency did. These studies show that EA-induced analgesic effect varies in different frequencies in the specific species. Acupoint is another important factor influencing EAA. Studies have demonstrated that EA stimulation at a set of Baihui and Santai acupoints can elicit an effective analgesia in goats [28, 31–33]. Zusanli acupoint has been widely used to induce analgesic effect in rats [8], rabbits [40], and humans [4]. In the present study, pain thresholds in goats receiving 60 Hz of EA at the set of Baihui and Santai acupoints and the set of bilateral Housanli acupoints for 30 min were increased by 44.74% ± 4.56% and 32.64% ± 5.04%, respectively, showing different analgesic effects induced by these two sets of acupoints.

Functional magnetic resonance imaging studies illustrated that the limbic and paralimbic structures of the cortical and subcortical regions in the telencephalon, the brainstem, and the cerebellum were excited after acupuncture was administered at bilateral Zusanli acupoints in human, showing that acupuncture-induced activated networks exist in the CNS [43]. However, the analgesia-related neural circuits activated by EA are not elucidated. Jang-Hern and Beitz [44] reported that EA of 4 Hz at bilateral Zusanli acupoints activated c-Fos-IR neurons in the PBN, LC, PAG, ARC, the habenula nucleus (HB), the substantia nigra (SNC), nucleus raphe pallidus, posterior pretectal nucleus, the cuneiform nucleus, the lateroventral and lateral hypothalamic nuclei, and SDH in rats, while EA of 100 Hz activated the PBN, LC, PAG, SNC, nucleus raphe pallidus, posterior pretectal nucleus, rostolateroventral nucleus of the medulla, and SDH. In our study, EA of 60 Hz at the set of bilateral Housanli acupoints activated the LSN, MSN, PVH, VMH, ARC, AB, HL, vlPAG, PBN, NRM, LC, GI, PG, and SDH. These studies suggest that EA specific circuits in the CNS might be mediated by different frequencies. Qiu et al. [31] indicated that EA of 60 Hz at Baihui, Santai, Ergen, and Sanyangluo acupoints induced increased c-Fos-IR neurons in the ACB, LSN, CAU, PVH, AB, ARC, HL, vlPAG, PBN, LC, NRM, NTS, GI, SNC, and the supraoptic nucleus in goats. We used EA of 60 Hz at the set of Baihui and Santai acupoints and found that the MSN, VMH, ARC, AB, HL, vlPAG, PBN, NRM, LC, GI, PG, and SDH were activated in goats. These show that the two sets of acupoints can activate more complex neural networks compared with one set of acupoints. Therefore, analysis of discrepancy in the activated cerebral nuclei or areas by EA at two sets of acupoints is useful for exploring the neural mechanism underlying EAA. Chae et al. [45] found that c-Fos-IR neurons activated by EA at bilateral Taiyuan acupoints were less than those by EA at bilateral Shenmen or Zusanli acupoints in the shell of ACB and that c-Fos-IR neurons induced by EA at bilateral Shenmen acupoints were less than those by EA at bilateral Taiyuan or Zusanli acupoints in the ventromedial of the striatum in repeated nicotine-induced behavioral sensitization rats. In the present study, c-Fos-IR neurons activated by EA at the set of Baihui and Santai acupoints were significantly different from EA at the set of bilateral Housanli acupoints in the MSN, PVH, AB, HL, and SDH. These studies suggest the cerebral nuclei and areas activated by EA are acupoint-dependent.

Researchers have done a lot of work to confirm the analgesia-related neural circuits with the stimulation and abolition of certain nerve fibres or nuclei as well as pharmacological approaches (assessing antagonists and antibodies). Several lines of evidence have shown that the PAG, one of the regions contributing to the endogenous pain inhibitory system [16, 46] and being involved in the analgesic effect of acupuncture [47, 48], projects to the RVM (mainly NRM) [49] and the LC [50]. Mokha et al. [51] identified that the NRM and LC mediated spinal nociceptive transmission through independent descending inhibitory pathways. Ao et al. [52] found that electroacupuncture could activate excitatory neurons in RVM. Several studies suggested that EA activated RVM might inhibit pain through a descending serotonergic system in the spinal cord [53, 54]. A classical study found that electrical stimulation of the LC caused a moderate attenuation [12] while the electrolytic lesion of the LC induced potentiation of acupuncture. These studies suggest that PAG-NRM/LC-SDH pathway constitutes the descending inhibitory pathway for EAA.

In addition, some studies have verified that pain regulation is involved in limbic system (AMY, HB, and ARC) midbrain (PAG) pathway. Functional MRI studies showed that the limbic system played a major role in regulation of EA analgesia [55–57]. The AMY sends neuronal projections to PAG [58–60] which in turn projects to analgesic modulating neurons in the RVM [49]. The study of Tershner and Helmstetter [61] suggests that AMY stimulation produces analgesia that is mediated in part by the release of opioid peptides within the ventral PAG. Microinjection of naloxone (an antagonist to opioid receptors) into the AMY can block EA-induced analgesia. [11]. Longchuan et al. demonstrated that the HB took part in the descending pathway of analgesia from ACB to PAG [62]. Wang et al. and Liu et al. found that the stimulation of HB induced an effect antagonist to the acupuncture analgesia through the inhibitory action in NRM. The ARC projects to PAG and LC [63–66]. Yin et al. reported that stimulation of the ARC significantly increased EAA and decreased EA-induced responses of neurons in the LC, which was reversed by i.p. injection of naloxone [67]. In present study, we found that the ARC, vlPAG, NRM, LC, and SDH were activated by EA at the set of Baihui and Santai or bilateral Housanli acupoints. It suggests that ARC-PAG-NRM/LC-SDH pathway is the common neural pathway activated by the two sets of acupoints. However, compared with EA at the set of bilateral Housanli acupoints, EA at the set of Baihui and Santai acupoints induced c-Fos-IR neurons to increase in AB, but to decrease in HL, suggesting different nuclei and areas of the limbic system can be activated by EA at different acupoints.

Several findings have shown that the hypothalamus-pituitary system takes part in EA. Pan et al. [68] found that the low frequency electrical stimulation of the Zusanli acupoints caused marked expression of c-fos in the PG, as well as in the ARC and some nearby hypothalamic nuclei. Cho et al. demonstrated with fRMI that the hypothalamus-pituitary system was excited after acupuncture and suggested a hypothesis that acupuncture analgesia is involved in the hypothalamus-pituitary system [69, 70]. In present study, the increased expression of c-Fos was induced by EA at the set of Baihui and Santai acupoints and the set of bilateral Housanli acupoints in some hypothalamus nuclei (ARC and VMH) and the PG. The results of the study provide additional evidence for participation of the hypothalamus-pituitary in EA regulation.

C-Fos expression is influenced by many factors. In laboratory animal experiment, acupuncture [44, 71], nociceptive stimulations [71, 72], and other stresses (restraint, noise, etc.) [18, 73] have been reported to upregulate c-Fos expression in the spinal cord or brain areas. Ji et al. [71] compared the spinal c-Fos expressions caused by EA and the nociceptive stimulation and found that EA induced dense c-Fos-IR neurons in laminae III and IV whereas the nociceptive stimulation elicited significantly increased c-Fos-IR neurons in laminae I and II of the SDH in rats. In pathological conditions, c-Fos expression may be important for the development of a pain state as part of adaptive response of the spinal cord to nociceptive input [74]. Because EA can maintain homeostasis of the body [75, 76], it can suppress c-Fos expression in the SDH induced by the noxious stimulation, which has been demonstrated by numerous studies [72, 77, 78]. Pan et al. [79, 80] reported both EA and nociceptive stimulation increased c-Fos expression in PG and some hypothalamic nuclei, suggesting that there is probably a partial overlap of the central pathways caused by EA and noxious stimulation. In present study, all the animals were accustomed to the experimental conditions to reduce stress, and our results suggested the specific analgesia circuits induced by EA alone.

## 5. Conclusion

Potent analgesia was induced by EA stimulation at both the set of Baihui and Santai acupoints and the set of bilateral Housanli acupoints with stronger effect of the former than the latter. EA at the set of Baihui and Santai acupoints or bilateral Housanli acupoints induced more c-Fos-IR neurons in MSN, VMH, AB, ARC, HL, vlPAG, PBN, NRM, LC, GI, PG, and SDH. The ARC-PAG-NRM/LC-SDH and the hypothalamus-pituitary may be the common neural pathway activated by EA. The HB and AMY in the limbic system may be acupoint-dependent for EAA.

## Figures and Tables

**Table 1:** The number of c-Fos-like immunoreactive cells in the nuclei or areas after goats received EA at two sets of acupoints (mean ± SD, number × 10^−1^/field, *n* = 7).

Nuclei and areas	Blank control	Sham control	EA at Housanli	EA at Baihui-Santai
CAU	9.88 ± 2.08	8.23 ± 1.35	9.91 ± 1.65	8.40 ± 1.30
ACB	9.18 ± 0.99	8.09 ± 0.88^b^	10.14 ± 1.55^a^	10.96 ± 1.42^a^
LSN	12.90 ± 1.98^b^	12.33 ± 1.46^b^	15.70 ± 0.77^a^	14.60 ± 1.90
MSN	10.64 ± 1.64^c^	10.86 ± 1.86^c^	17.07 ± 1.45^a^	14.41 ± 1.71^b^
PVH	13.07 ± 0.89^b^	13.03 ± 1.76^b^	18.42 ± 1.94^a^	14.40 ± 1.85^b^
VMH	8.93 ± 0.78^b^	8.95 ± 0.86^b^	14.31 ± 1.05^a^	14.84 ± 1.74^a^
AB	8.50 ± 1.10^c^	8.27 ± 0.91^c^	11.61 ± 2.69^b^	15.35 ± 2.22^a^
ARC	16.72 ± 1.59^b^	17.27 ± 1.55^b^	22.20 ± 2.15^a^	23.17 ± 1.04^a^
HL	8.58 ± 0.90^c^	8.28 ± 1.84^c^	15.39 ± 2.34^a^	12.22 ± 0.75^b^
vlPAG	12.81 ± 0.62^b^	12.89 ± 1.37^b^	16.04 ± 0.88^a^	16.70 ± 0.68^a^
PBN	9.38 ± 1.06^b^	10.84 ± 1.08^b^	14.12 ± 0.96^a^	14.30 ± 1.29^a^
LC	9.31 ± 2.39^b^	10.16 ± 1.60^b^	13.65 ± 1.06^a^	15.27 ± 2.40^a^
NRM	6.68 ± 0.78^b^	7.19 ± 1.45^b^	11.56 ± 2.21^a^	11.15 ± 2.67^a^
NTS	12.40 ± 0.81	11.74 ± 1.99	12.34 ± 1.55	11.15 ± 1.33
GI	4.35 ± 1.70^b^	4.78 ± 1.65	7.91 ± 0.95^a^	7.88 ± 2.04^a^
PG	30.37 ± 14.23^b^	30.56 ± 8.31^b^	51.23 ± 4.43^a^	49.79 ± 10.14^a^
SDH	8.21 ± 1.18^c^	8.96 ± 0.93^c^	14.12 ± 0.92^a^	12.35 ± 0.94^b^

Values with different letters in the same row differ significantly (*p* < 0.05). The blank control refers to the group in which the goats were restrained as the EA-treated goats without needling and electric stimulation. The sham control refers to the group in which needles were inserted into Baihui and Santai acupoints of the goats without electric stimulation. The nuclei and areas are presented as follows: the caudate nucleus (CAU), the nucleus accumbens (ACB), the lateral septal nucleus (LSN), the medial septal nucleus (MSN), the paraventricular nucleus of the hypothalamus (PVH), the ventromedial nucleus of the hypothalamus (VMH), the arcuate nucleus (ARC), the nucleus amygdala basalis (AB), the lateral habenula nucleus (HL), the ventrolateral periaqueductal grey (vlPAG), the parabrachial nucleus (PBN), the locus coeruleus (LC), the nucleus raphe magnus (NRM), the gigantocellular reticular nucleus (GI), the nucleus tractus solitarius (NTS), the anterior lobe of the pituitary gland (PG), and spinal cord dorsal horn (SDH).

## Abbreviations

AB: The nucleus amygdala basalis
ACB: The nucleus accumbens
AMY: The nucleus amygdala
ARC: The arcuate nucleus
CAU: The caudate nucleus
C-Fos-IR: c-Fos-like immunoreactive
CNS: The central nervous system
EA: Electroacupuncture
EAA: Electroacupuncture-induced analgesia
fMRI: Functional magnetic resonance imaging
GI: The gigantocellular reticular nucleus
HB: The habenula nucleus
HL: The lateral habenula nucleus
LC: The locus coeruleus
LSN: The lateral septal nucleus
MSN: The medial septal nucleus
NRM: The nucleus raphe magnus
NTS: The nucleus tractus solitaries
PAG: The periaqueductal grey
PBN: The parabrachial nucleus
PG: The anterior lobe of the pituitary gland
PVH: The paraventricular nucleus of the hypothalamus
RVM: Rostral ventromedial medulla
SDH: Spinal cord dorsal horn
SN: The septal nucleus
SNC: The substantia nigra
vlPAG: The ventrolateral periaqueductal grey
VMH: The ventromedial nucleus of the hypothalamus.
